# Ligation and mucopexy for prolapsing hemorrhoids – a ten year experience

**DOI:** 10.1186/1750-1164-2-5

**Published:** 2008-11-28

**Authors:** Pravin J Gupta, Surekha Kalaskar

**Affiliations:** 1 Fine Morning Hospital and Research Center Gupta Nursing Home, Laxminagar, Nagpur-440022, India

## Abstract

**Objective:**

The aim of this study is to clinically test the efficacy of author's approach of suture ligation and mucopexy for patients having symptomatic and prolapsing hemorrhoids.

**Materials and methods:**

616 patients (255 females) complaining of symptoms of hemorrhoids were included in the study. The hemorrhoids were suture ligated with an absorbable suture material under vision. Operating time, postoperative complications, time to return to work, and outcome of the procedure were analyzed. Follow-up was planned following discharge after 1 month, 6 months and after at least 1 year. Patient satisfaction was also assessed.

**Results:**

The mean procedure time was 8 ± 0 minutes (range, 6–15 minutes), and the total admission period was 12 ± 4 Hours. Perianal thrombosis and skin tags were the commonest post-operative complications. The mean total analgesic dose and duration of pain control using analgesics was 19 ± 4 tablets, and 9 ± 3 days respectively.

The postoperative follow up after 4 weeks revealed therapeutic success in 589 patients (95.6%), who presented with hemorrhoidal bleeding. Prolapse was no longer observed in 98% of patients and 96% patients experienced no pain after defecation. 93% patients completed the one-year follow-up and 89 percent of them were asymptomatic. The patient satisfaction scoring was 8.2% on visual analogue scale.

**Conclusion:**

Suture ligation and mucopexy of hemorrhoids is an easy-to-perform technique that is well accepted by patients and has good results for prolapsing hemorrhoids.

## Background

Many therapeutic options exist for the treatment of symptomatic hemorrhoids; among them are dietary and lifestyle modifications and office treatment such as infrared coagulation, sclerotherapy, rubber band ligation, hemorrhoidal artery ligation, or various stapling and excisional procedures [1].

We describe a simpler technique for the reduction of the size of the hemorrhoids with control of bleeding and prolapse, which we term as ligation and mucopexy of the hemorrhoids under vision. This technique is based on the fact that the hemorrhoidal vessels have a constant anatomical location. Usually, they penetrate the hemorrhoid pile in the base. A stitch that is put on the base of the hemorrhoid cushion is able to significantly diminish the blood flow to the hemorrhoidal plexus. And along with the base, if complete ligation of all the visual hemorrhoidal cushions is performed, it will control the prolapse as well. This procedure of ligation of hemorrhoidal cushion has a long history and is termed with various nomenclatures like 'pile suture' [2], 'obliterative suture technique' [3], 'ligation and anopexy' [4] and 'suture ligation' [5] etc.

The pile suture technique practiced by Farag, and its modifications proposed by others, have failed to gain wide acceptance, because they were directed mainly at reduction of blood flow to the hemorrhoidal cushions, which was associated with initial painful congestion followed by gradual shrinkage of prolapsed hemorrhoids.

Our procedure of ligation and mucopexy is designed to restore fixation of the hemorrhoidal cushions to the underlying internal sphincter to reduce hemorrhoidal prolapse in addition with minimizing the blood flow to the plexus. This minimally invasive ambulatory procedure is developed as an option for patients who are candidates for operative hemorrhoidectomy. The rationale of the technique is based on the fact that hemorrhoidal prolapse is the result of sliding down of the anal mucosa caused by attenuation of the anchoring elastic tissue system and fixing this sliding mucosa will restore the cushion back to its original position [6].

This study describes the surgical procedure and assesses its benefits in terms of surgical outcome, functional recovery, and postoperative pain in a relatively large patient cohort. This report also evaluates the results of this procedure over the past ten years in the treatment of prolapsing hemorrhoidal disease.

## Materials and methods

Patients having symptomatic and prolapsing hemorrhoids attending the outpatient clinic were included in this study. Exclusion criteria were acutely thrombosed piles or concurrent anal pathology (e.g., fistula, fissures etc). Patients suffering from Grade II hemorrhoids were considered suitable for surgery because of the severity of symptoms (i.e., profuse bleeding) despite previous conventional less invasive treatments, and also were scheduled for surgery at their own specific request.

The patients underwent a complete medical history with emphasis on hemorrhoidal symptoms, previous conservative or surgical treatment, and other anorectal condition. Clinical examination, anoscopy, rigid rectoscopy, and colonoscopy were performed to accurately stage the disease and rule out other colorectal conditions in every patient older than 50 years. The four outcome measures were symptomatic recurrence, post procedure pain, incidence of complications, and patient satisfaction after at least 12-month post procedure.

### Surgical procedure

**Figure 1:**
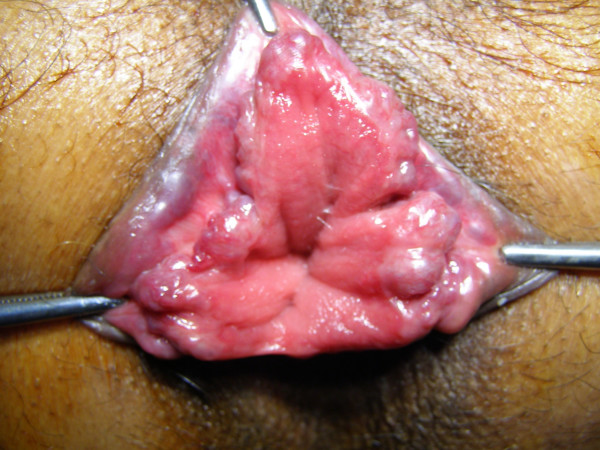
Hemorrhoids at multiple positions.

**Figure 2:**
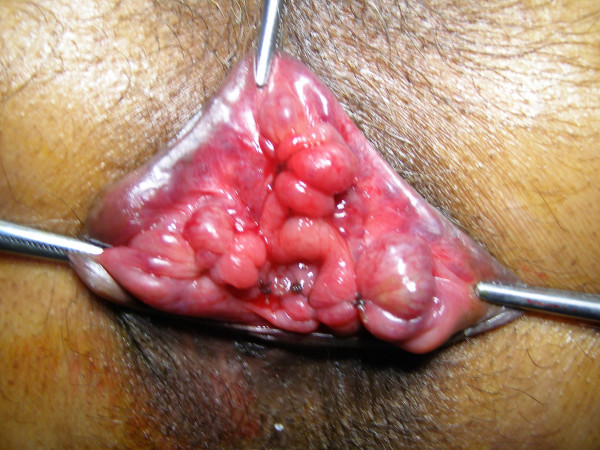
Hemorrhoids after ligation and mucopexy.

With the patient in a lithotomy position, the three skin tags corresponding to three principle sites of hemorrhoidal cushions, namely 3, 7 and 11'O clock position were held with artery forceps and retracted out to visualize the hemorrhoids. The hemorrhoidal cushion was then sutured using a half-circle 45 mm round needle and absorbable 1-0 chromic catgut (No. 4246 Ethicon UK). Firstly, a transfixing suture was applied at the hemorrhoidal pedicle. A new suturing began caudally in a continuous locking manner and included the mucosa, submucosa and half the depth of the anal sphincter muscles to end just 5 mm below the dentate line. The ligations were performed above the dentate line in a relatively insensitive region. Any secondary hemorrhoids found were also treated on the same line as the primary hemorrhoids [Figure 1 and 2].

The patients were assessed after 8 hours of the procedure and were discharged if they were found comfortable with regard to pain and reporting no difficulty in passing urine. All the patients were prescribed with a combination of Tramadol Hydrochloride and Paracetamol for post procedure analgesia. They were instructed to take these tablets as and when required and to attend the casualty department whenever the pain was intolerable or any significant complications developed, especially spontaneous bleeding or perianal sepsis. Home treatment also included a high-residue diet, stool softeners, and immediate warm sitz baths. Patients were instructed to assess the postoperative pain using a 10-cm linear analog scale in which 0 corresponds to "no pain" and 10 to "maximum pain" and to record the use of analgesics on the day of surgery and everyday for the first 14 days. Our advice was to return to work and normal daily activities as soon as they felt able to do so. The patients were followed-up at 4 weeks, 6, and 12 months and anytime afterward if they have any complaints. Patient satisfaction rate was assessed at the 12-month follow-up visits using a 10-point scale (1 = extremely dissatisfied, 10 = very satisfied).

## Results

From May 1997 through December 2007, 616 patients were treated by this technique in our clinic. The group consisted of 361 male patients and 255 females (age 11 to 93 years). Main symptoms presented were bleeding and prolapsing hemorrhoids. The median duration of symptoms was 6.3 years.

The procedure was performed in 76 patients with grade II, 441 patients with grade III and 99 patients with grade IV hemorrhoids. Patients were treated either under local, spinal or short-term general anesthesia as per the decision of the anesthesiologist.

The total admission period was 12 ± 4 Hours. On average, 3.12 hemorrhoids were suture ligated per patient. The mean procedure time was 8 ± 0 minutes (range, 6–15 minutes).

The mean total analgesic dose and duration of pain control using analgesics was 19 ± 4 tablets, and 9 ± 3 days respectively.

Complications were identified in 9% patients, which included retention of urine; pain needing readmission, bleeding needing readmission, external hemorrhoidal thrombosis, anal tags and pruritus [Table 1].

**Table 1:** Complications after ligation and mucopexy of hemorrhoids. [Total number of patients 616]

Complications	Number of patients
Perianal thrombosis	12
Bleeding needing readmission	4
Pain needing readmission	2
Urinary retention	9
Pruritus ani	2
Mucosal prolapse	6
Skin tag	13
Constipation	4
Tenesmus	4

The postoperative follow up after 4 weeks revealed therapeutic success in 589 patients (95.6%), who presented with hemorrhoidal bleeding. Prolapse was no longer observed in 98% of patients and 96% patients experienced no pain after defecation.

93% patients completed the one-year follow-up and 89 percent of them were asymptomatic. No anal narrowing was observed in any patient during rectal digitation.

Up to May 2006, a total of 485 patients were treated with this method. 307 patients responded to our inquiry conducted at the beginning of 2007. Ninety-four percent of these patients confirmed that they no longer experienced any bleeding or pain during defecation and eighty-nine percent patients did not had any prolapse. On rectal examination of the remaining 11% of patients complaining of prolapse, it was found that four percent of them had residual skin tags which they were considering as prolapse. They were reassured about the benign nature of these tags. The patients complaining of intermittent bleeding were treated conservatively with flavonide derivatives, stool softeners and dietary modification. Those not responding to these measures were treated with band ligation or infra red coagulation. Those complaining of prolapse were offered a redo procedure.

Seven patients complained of continence disturbances. As a preoperative anal sphincter function assessment was not conducted, we were unable to know whether this anal sphincter dysfunction was already present preoperatively and if the symptoms of incontinence were compensated for by the prolapsing hemorrhoids, as we do not see a direct relation to the procedure as such.

The stress induced by the procedure was considered to be minor by 89% of patient and the satisfaction scoring was 8.2% on visual analogue scale.

## Discussion

Hemorrhoids are described as a plexus of veins located between the lamina muscularis mucosa and sphincter muscle structures. This plexus is supported by elastic tissue and the muscular structure of Treitz (m.canalis ani) and consists of a superior (inner) and inferior (external) part divided by the dentate line. Because of arterial shunts and an extension of veins, this plexus becomes enlarged and plays an important role in fine continence of the anal canal [7]. The system, which is also called the "corpus cavernosum recti", is complex, and some of its functions are still unknown. Hemorrhoids are physiologic and represent a part of the anal sphincter system. However, nonphysiologic enlargement and displacement of this anorectal plexus, together with symptoms must be considered a disease [8].

Excisional hemorrhoidectomy is usually reserved for complicated cases and for patients who do not respond to the instrumental office procedures. Stapled hemorrhoidectomy by means of a transanal stapled technique requires a specially designed circular stapler [9]. The outcome after stapler hemorrhoidectomy does not depend solely on the complete interruption of the arterial inflow of the hemorrhoids, and flow can still be detected by Doppler ultrasound in the main branches of the superior rectal artery in as many as 80 percent of the patients at one month postoperatively [10]. Some studies even assigned the course of the arterial branches to exactly defined positions in the rectal submucosa (3'0, 7'0 and 11'0, as viewed in the anatomic lithotomy position) corresponding to the clinical appearance of hemorrhoids [11].

While considering any interventional technique to tackle the hemorrhoids, it must be remembered that the corpus cavernosum recti plays an important role within the anal canal and there is a fine line between successful treatment and the risk of damaging the anal sphincter [12].

The procedure described by us can be termed as a minimally invasive as it does not involve any mucosal or anodermal excision and is very simple to perform as it follows a very basic surgical maneuver, i.e., suturing. The goal of the treatment is to reduce the blood supply to the hemorrhoidal plexus and thus to shrink the hemorrhoids. As the arteries carrying the blood inflow are ligated, the internal pressure of the plexus hemorhoidalis is decreased and the typical symptoms of hemorrhoids disappear [13]. During the postoperative follow-ups, the treated hemorrhoids by our technique were found to be replaced by segmented fibrotic scar firmly adhered to the underlying structures.

We believe that ligation and mucopexy of hemorrhoids is relatively painless, easily learned and minimally invasive therapeutic technique that offers a good alternative to all other known treatment of symptomatic and prolapsing hemorrhoids. There is always the possibility of revascularization and recurrence of symptoms of hemorrhoids, but this procedure can be repeated at any time [14].

It has been suggested that the source of remnant or secondary hemorrhoids is from the unobliterated vessels, which are present on the posterolateral position of the rectal wall [15]. Suture ligation ably takes care of these vessels too. As suture-ligation is confined to the protruding hemorrhoids only and does not attempt any excision, it preserves the sensitive anoderm and the rectal mucosa. Thus, this study shows that ligation and mucopexy of the hemorrhoids decreases arterial inflow and achieves fixation of the hemorrhoidal cushions. The complications are comparable with those associated with other methods and no complications of serious nature were observed.

## Conclusion

The procedure described by us for prolapsing hemorrhoids is associated with less pain than historic controls, shorter recovery period, and low complication rate, and is well-tolerated by the patients. Most of our patients were operated on using an ambulatory setting and were discharged after short observation, and most of them returned to normal daily activities by the tenth postoperative day.

## Consent

Written informed consent was obtained from the patient for publication of this report and accompanying images.

## Competing interests

The authors declare that they have no competing interests.

## Authors' contributions

PJG carried out the surgical procedures and SK carried out the pre-operative assessment of the patients, data collection and compilation. Both the authors read and approved the final manuscript.
